# Correction: Relevance of tumor boards for the inclusion of patients in oncological clinical trials

**DOI:** 10.1007/s00432-024-06061-1

**Published:** 2025-01-18

**Authors:** Hendrik Dapper, Maurice Dantes, Peter Herschbach, Hana Algül, Volker Heinemann

**Affiliations:** 1 Comprehensive Cancer Center Munich, Klinikum rechts der Isar, Technical University Munich, Munich, Germany; 2 Department of Radiation Oncology, Comprehensive Cancer Center Munich, University Hospital Munich, LMU Munich, Munich, Germany; 3 Department of Internal Medicine II, Comprehensive Cancer Center Munich, Klinikum rechts der Isar, Technical University Munich, Munich, Germany; 4 Comprehensive Cancer Center München, Institute for Tumor Metabolism, Klinikum rechts der Isar, Technical University of Munich, Munich, Bavaria Germany

**Correction: Journal of Cancer Research and Clinical Oncology (2023) 149:7601-7608**

10.1007/s00432-022-04559-0

In this article the affiliation details for author Hana Algül were incorrectly given as ‘Department of Internal Medicine II, Comprehensive Cancer Center Munich, Klinikum rechts der Isar, Technical University Munich, Munich, Germany’ but should have been ‘Comprehensive Cancer Center München, Institute for Tumor Metabolism, Klinikum rechts der Isar, Technical University of Munich, Munich, Bavaria, Germany’.

The content of the article remains unchanged.

The original article has been corrected.

